# Myeloma Precursors Erode Durable Immunity: Results of the IMPACT study

**DOI:** 10.21203/rs.3.rs-9226435/v1

**Published:** 2026-04-14

**Authors:** Noé Perron, Michelle P. Aranha, Ting Wu, Lorena P. Rubino, Hong Yue, Elizabeth D. Lightbody, Riddhiman Saha, Mahshid Rahmat, Michael Timonian, Daniel Heilpern-Mallory, Michael P. Agius, Saveliy Belkin, Radoslaw P. Nowak, Jacqueline Perry, Erica Horowitz, Mark Hamilton, Daniel Auclair, Hearn J. Cho, George Mulligan, Eric S. Fischer, Omar Nadeem, Catherine Marinac, Lorenzo Trippa, Gad Getz, Romanos Sklavenitis-Pistofidis, Irene M. Ghobrial, Yoshinobu Konishi

**Affiliations:** 1 Department of Medical Oncology, Dana-Farber Cancer Institute, Boston, MA, USA.; 2 Center for Early Detection and Interception of Blood Cancers, Dana-Farber Cancer Institute, Boston, MA, USA.; 3 Department of Medicine, Harvard Medical School, Boston, MA, USA.; 4 Broad Institute of Massachusetts Institute of Technology (MIT) and Harvard, Cambridge, MA, USA.; 5 Cancer Center and Department of Pathology, Massachusetts General Hospital, Boston, MA, USA.; 6 Department of Cancer Biology, Dana-Farber Cancer Institute, Boston, MA, USA.; 7 Department of Biological Chemistry and Molecular Pharmacology, Harvard Medical School, Boston, MA, USA.; 8 Department of Data Science, Dana-Farber Cancer Institute, Boston, MA, USA.; 9 Department of Biostatistics of Harvard TH Chan School of Public Health, Boston, MA, USA.; 10 Multiple Myeloma Research Foundation (MMRF), Norwalk, CT, USA.

## Abstract

Multiple myeloma (MM) is a plasma cell malignancy marked by profound immune dysfunction and substantial infection-related mortality. MM is preceded by the asymptomatic precursor states monoclonal gammopathy of undetermined significance (MGUS) and smoldering multiple myeloma (SMM), yet whether these patients can mount effective immune responses remains unknown. In this prospective observational study of 731 individuals recruited nationwide through Dana-Farber Cancer Institute, we used SARS-CoV-2 vaccination as a standardized immunological challenge alongside childhood vaccine serology. We show that precursor myeloma is associated with broad dysfunction across innate and adaptive immunity, including accelerated antibody waning, erosion of childhood vaccine titers, impaired antigen-specific T cell expansion, and blunted innate activation. Mechanistically, we link these defects to tumor burden-dependent APRIL depletion and downregulation of APRIL-responsive pathways in normal plasma cells, validated in an independent cohort. These findings have direct implications for cancer vaccine development, immunotherapy, and infection management in early-stage hematologic malignancies.

## INTRODUCTION

The emergence of therapeutic cancer vaccines, together with vaccines against infectious diseases such as SARS-CoV-2, represents a major advance in modern medicine. Progress in neoantigen discovery, mRNA delivery platforms and immune checkpoint modulation has accelerated the development of personalized vaccines targeting tumor-specific alterations^1–3^. Early clinical trials have demonstrated encouraging responses in melanoma^4^, pancreatic cancer^5,6^, and other solid tumors, establishing that the immune system can be harnessed to recognize and eliminate cancer cells through vaccination^1^. More broadly, immune competence is a critical determinant of protection against infection, which remains a major cause of morbidity and mortality in patients with cancer, particularly those with hematologic malignancies.

Multiple myeloma (MM) is a plasma cell malignancy marked by profound immune dysfunction. During the COVID-19 pandemic, patients with active MM had poor outcomes, with mortality driven more often by infection than by treatment-related toxicity or disease progression^7^. MM is therefore a particularly informative model in which to examine vaccine responsiveness, as the disease itself arises within and progressively reshapes the immune system. Defining the immunologic substrate on which both protective immunity and therapeutic vaccination depend is thus of central importance.

MM is preceded by the asymptomatic precursor conditions monoclonal gammopathy of undetermined significance (MGUS) and smoldering multiple myeloma (SMM), which affect an estimated 3–5% of adults older than 50 years, with prevalence increasing with age, while most affected individuals remain undiagnosed^8–10^. These precursor states provide a unique window into how an evolving malignancy perturbs immune function before the onset of symptomatic disease. Prior studies have identified reduced T cell diversity, depletion of granzyme K–positive memory cytotoxic T cells, altered myeloid populations and compromised humoral immunity in MGUS and SMM, indicating that immune dysregulation is already established during the precursor phase^11–13^. Consistent with this, individuals with MGUS and SMM have an increased risk of bacterial and viral infections compared to the general population, even in the absence of treatment-related immunosuppression^14^. Together, these observations define a large population of aging individuals with occult immune dysfunction who are increasingly being considered for early therapeutic intervention, including cancer vaccination^15^, yet whose capacity to mount effective immune responses to vaccination remains poorly understood.

To address this, we established the IMPACT (IMmune Profiling And COVID-19 Trajectory) study, a prospective observational cohort of 731 participants, including healthy donors and patients with MGUS, SMM and overt MM, recruited through Dana-Farber Cancer Institute during the SARS-CoV-2 pandemic. Using SARS-CoV-2 mRNA vaccination as a standardized challenge to a novel antigen, together with measurement of antibodies to measles, mumps and rubella (MMR) vaccines administered decades earlier, we distinguished defects in de novo immune priming from defects in long-term plasma cell maintenance. Integrated immune profiling included 1,940 longitudinal antibody measurements, single-cell RNA sequencing, single-cell T cell receptor sequencing, plasma proteomics and functional T cell assays.

We show that myeloma precursor conditions are characterized by broad immune hyporesponsiveness that begins during the clinically silent phase of disease. Patients exhibited accelerated waning of antibody responses to SARS-CoV-2 vaccination and reduced titers to childhood MMR vaccines, implicating impaired maintenance of humoral immunity. These defects were associated with tumor burden–dependent depletion of circulating APRIL and downregulation of key APRIL-responsive survival pathways in patient’s plasma cells, findings validated in an independent cohort of bone marrow plasma cells. In parallel, patients failed to mount de novo antigen-specific T cell responses after vaccination, while innate cytokine and pattern-recognition receptor responses were broadly attenuated. Together, these findings define a multilayered immune deficit in precursor myeloma with direct implications for vaccine-based immunotherapy and infection management in early-stage hematologic malignancies.

## RESULTS

### Accelerated antibody waning with disease progression in myeloma precursors

We previously demonstrated that patients with SMM exhibit significantly lower spike-specific IgG antibody levels following two doses of SARS-CoV-2 vaccination compared to healthy donors, with antibody levels inversely correlating with disease burden^16^. To define the temporal dynamics of this defect and extend these findings to MGUS/SMM, we initiated the IMPACT (IMmune Profiling And COVID-19 Trajectory) study (DFCI Protocol 20–332), a prospective observational study conducted nationwide and samples were analyzed at Dana-Farber Cancer Institute (DFCI), Boston, MA. The study was designed to characterize immune responses to vaccination in patients with myeloma precursor conditions and matched controls. Participants were enrolled between November 2020 and April 2022. Participants were healthy controls (HD), or patients diagnosed with MGUS, SMM, or MM and received at least one dose of an FDA-authorized SARS-CoV-2 mRNA vaccine (BNT162b2, Pfizer-BioNTech, or mRNA-1273, Moderna). Baseline characteristics are described in Supplementary Table 1.

**Figure 1.**
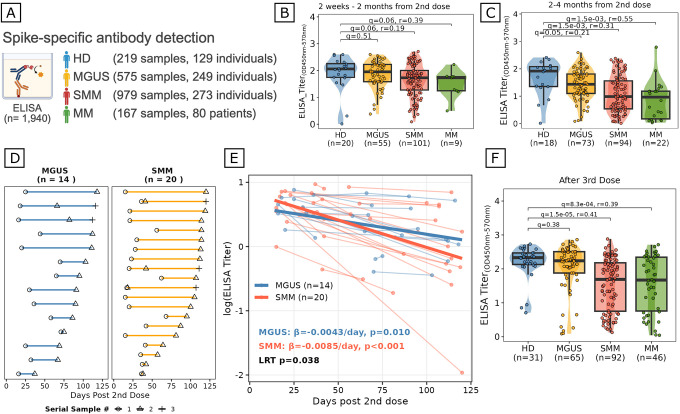
Impaired maintenance of humoral immunity in myeloma precursor conditions. (A) Schematic overview of the ELISA cohort. Spike-specific IgG antibody titers were measured in 1,940 plasma samples from 731 individuals across disease stages: healthy donors (HD; n=219 samples, 129 individuals), MGUS (n=575 samples, 249 individuals), SMM (n=979 samples, 273 individuals), and MM (n=167 samples, 80 individuals) (created with BioRender). (B-C) Spike-specific antibody titers measured by ELISA in samples collected 2 weeks to 2 months (B) or 2 to 4 months (C) after the second vaccine dose. Sample sizes: (B) HD, n=20; MGUS, n=55; SMM, n=101; MM, n=9; (C) HD, n=18; MGUS, n=73; SMM, n=94; MM, n=22. Violin plots show density distribution; boxes indicate median and interquartile range (IQR); whiskers extend to 1.5xIQR. Statistical comparisons to HD were performed using two-sided Wilcoxon rank-sum tests with Benjamini-Hochberg (BH) correction for multiple testing (3 tests per panel). Effect sizes are reported as rank-biserial correlation (r) for comparisons reaching q<0.1. (D) Swimmer plot showing longitudinal sampling timeline for patients with MGUS (n=14) and SMM (n=20) who had serial post-vaccination samples collected. Each horizontal line represents an individual patient; symbols indicate sample collection timepoints with shapes denoting sample order (circle=1st, triangle=2nd, cross=3rd). (E) Linear mixed-effects modeling of antibody waning rates in patients with MGUS (n=14) and SMM (n=21) who had >=2 serial samples collected between 14–120 days post-second dose. Individual patient trajectories are shown as thin lines; bold lines represent model-fitted group-level slopes. Waning rates (β, log-titer change per day) and p-values for each group are indicated. The likelihood ratio test (LRT) comparing models with disease-specific versus common slopes demonstrates significantly faster waning in SMM compared to MGUS (p=0.040). (F) Spike-specific antibody titers after the third vaccine dose. Sample sizes: HD, n=31; MGUS, n=65; SMM, n=92; MM, n=46. Plot elements and statistical methods as in (B-C).

We measured spike-specific IgG levels by ELISA in 1,940 samples collected pre- and post-vaccination from 731 individuals, including HD (n=129), patients with MGUS (n=249), SMM (n=273), and overt MM (n=80) (Figure 1A). At peak response (2 weeks to 2 months post second dose^17,18^), SMM and MM patients showed lower spike-specific IgG levels compared to HD (q=0.06 for both), with a moderate effect size for MM (r=0.39) and a smaller effect for SMM (r=0.19), while MGUS titers were not significantly different from HD (q=0.51; Figure 1B). In contrast, at later timepoints (2–4 months post second dose), the gap widened substantially: MGUS (q=0.05, r=0.21), SMM (q=1.5×10–3, r=0.31), and MM (q=1.5×10–3, r=0.55) all showed reduced titers compared to HD, with effect sizes increasing along the disease continuum (Figure 1C). This widening gap and increasing effect sizes over time suggest a defect not in the initial generation but in the maintenance of the antibody response.

To directly quantify the rate of antibody decline, we collected serial post-vaccination samples from patients with MGUS (n=14) and SMM (n=20) and modeled individual titer trajectories using a linear mixed-effects framework with patient-level random intercepts (Figure 1D–E). This analysis revealed that spike-specific antibodies declined approximately twice as fast in patients with SMM compared to MGUS (β=−0.0085 vs. −0.0043 log-titer/day; likelihood ratio test, p=0.038), demonstrating that the rate of antibody waning accelerates along the MGUS-to-SMM disease continuum. Importantly, this accelerated waning was not overcome by additional vaccination: even after a third dose, SMM and MM patients continued to have significantly lower spike-specific titers compared to HD (SMM: q=1.5×10–5, r=0.41; MM: q=8.3×10–4, r=0.39; Figure 1F). Despite the absence of symptoms and overt disease, patients with myeloma precursors, and particularly those with SMM, exhibit accelerated waning of vaccine-induced antibody titers that cannot be simply overcome by boosting, pointing to a fundamental defect in the maintenance of humoral immunity.

### Erosion of long-term immunity and APRIL depletion in myeloma precursors

**Figure 2.**
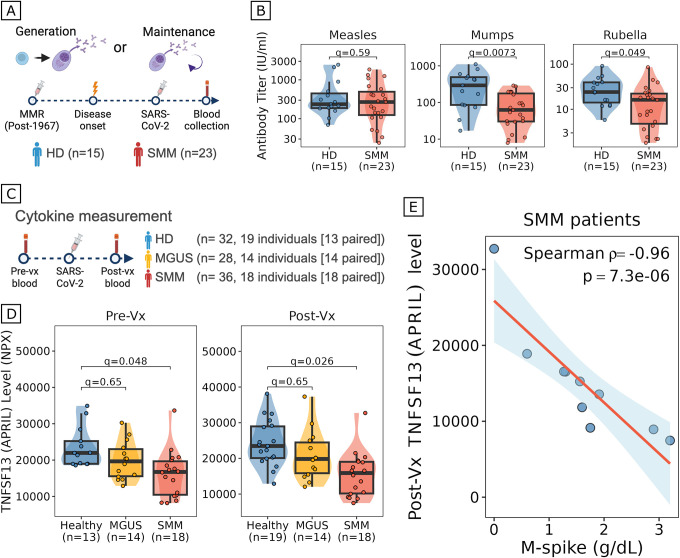
Erosion of long-established immunity and APRIL depletion in myeloma precursors. (A) Schematic of experimental design (created with BioRender). Anti-MMR IgG antibodies were measured in peripheral blood plasma from individuals born after 1967, when childhood MMR vaccination became widespread in the United States (HD, n=15; SMM, n=23). (B) Box plots, violin plots, and scatter plots comparing anti-measles, anti-mumps, and anti-rubella IgG antibody levels between HD (n=15) and patients with SMM (n=23). Violin outline width represents density. Box: median and interquartile range (IQR); whiskers: 1.5×IQR. Each dot represents one individual. q-values were computed with two-sided Wilcoxon rank-sum tests and corrected using the Benjamini-Hochberg (BH) method. (C) Schematic of Olink-based plasma proteomics experimental design (created with BioRender). Cytokine levels were measured in paired pre- and post-vaccination peripheral blood plasma from HD (n=32 samples from 19 individuals, 13 with paired samples), MGUS (n=28 samples from 14 individuals, all paired), and SMM (n=36 samples from 18 individuals, all paired). (D) Box plots, violin plots, and scatter plots comparing peripheral blood plasma APRIL (TNFSF13) levels (y-axis, Olink NPX) pre-vaccination (left) and post-vaccination (right) in HD (pre-Vx, n=13; post-Vx, n=19), MGUS (n=14), and SMM (n=18). Violin outline width represents density. Box: median and IQR; whiskers: 1.5×IQR. Each dot represents one individual. q-values were computed by comparing each disease stage to HD with two-sided Wilcoxon rank-sum tests and corrected using the BH method. (E) Scatter plot of the correlation between serum M-protein levels at baseline (x-axis) and post-vaccination plasma APRIL levels (y-axis) in patients with SMM. The Spearman correlation coefficient and two-sided p-value are shown. A regression line was fit using the linear model method and the confidence interval is shown in blue shading.

Because antibody titer generation is preserved to a degree in patients with SMM and because antibody titers wane progressively faster in patients of more advanced stage, we hypothesized that the observed defect could reflect more of an impairment in plasma cell maintenance rather than generation. We reasoned that antibody responses to antigens encountered during childhood or young adulthood, long before plasma cell (pre-)malignancy is established (which is estimated to occur in the ~3rd decade of life), should be unaffected by a disease-driven defect in antibody generation but impacted by a disease-driven defect in antibody maintenance^19–21^. To deconvolve antibody generation from antibody maintenance, we measured antibody levels for Measles, Mumps, and Rubella (MMR) using ELISA in individuals born after 1967, when MMR vaccination became broadly available in the US (Figure 2A). We observed significantly lower levels of antibodies against Mumps and Rubella in patients with SMM (n=23), compared to HD (n=15) (two-sided Wilcoxon, Measles: p=0.595; Mumps: p=0.007; Rubella: p=0.049), confirming that patients with SMM exhibit a defect in plasma cell maintenance that affects both recent and past vaccine-induced immune responses (Figure 2B).

Long-lived plasma cells critically depend on the survival factor APRIL (encoded by *TNFSF13*), which is produced by stromal and myeloid cells in the bone marrow niche and signals notably through the BCMA receptor on plasma cells^22–26^. We hypothesized that expanding clonal plasma cells in myeloma precursors might act as a “sink” for circulating APRIL, progressively sequestering this survival factor and thereby depriving normal antibody-secreting cells of a critical maintenance signal. To test this hypothesis, we performed plasma cytokine profiling using Olink-based proteomics on paired pre- and post-vaccination samples from 51 individuals (HD n=19, MGUS n=14, SMM n=18; Figure 2C). Strikingly, plasma APRIL levels were significantly reduced in SMM patients compared to healthy donors at both pre-vaccination (q=0.048) and post-vaccination (q=0.026) timepoints, with MGUS patients displaying intermediate levels consistent with a disease stage-dependent effect (Figure 2D). Notably, APRIL levels were comparably reduced before and after vaccination, indicating that APRIL depletion reflects an underlying disease-associated state rather than a consequence of vaccination itself. If APRIL depletion reflects consumption by expanding clonal plasma cells, we would expect APRIL levels to inversely correlate with tumor burden. Indeed, in SMM patients, post-vaccination APRIL levels showed a strong inverse correlation with M-spike, a measure of tumor burden (Spearman ρ=−0.96, p=7.3 10^−6^; Figure 2E), consistent with our hypothesized model of APRIL depletion by expanding clonal plasma cells.

### Impaired APRIL signaling in normal B cells from patients with myeloma precursors

**Figure 3.**
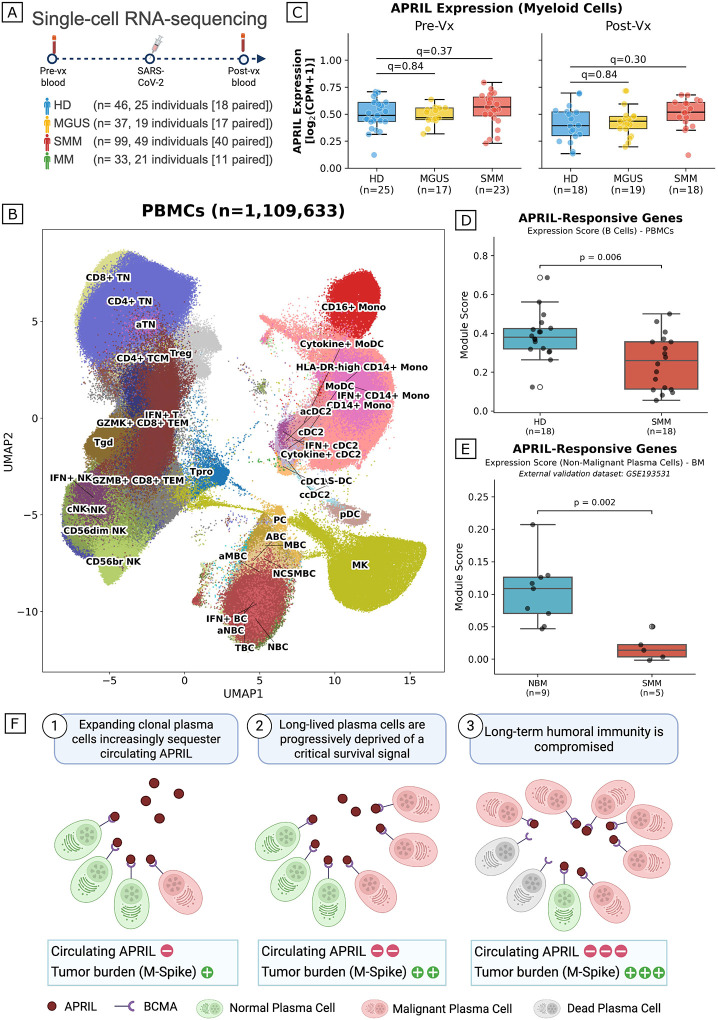
Impaired APRIL signaling in myeloma precursor B cells is consistent with tumor-driven consumption. (A) Schematic of the single-cell RNA sequencing (scRNA-seq) experimental design (created with BioRender). scRNA-seq was performed on peripheral blood mononuclear cells (PBMCs) from 114 individuals across the disease spectrum (HD, n=25; MGUS, n=19; SMM, n=49; MM, n=21), with pre- and post-vaccination samples collected. (B) Uniform manifold approximation and projection (UMAP) embedding of 1,109,633 cells, including T cells, B cells, NK cells, monocytes, dendritic cells, and progenitor cells, colored by and labeled with cell subtype annotations. A total of 37 distinct cell populations were identified through unsupervised clustering and manual annotation. (C) Box plots comparing TNFSF13 (APRIL) transcript expression in myeloid cells (monocytes and dendritic cells) between HD, MGUS, and SMM at pre-vaccination (left) and post-vaccination (right) timepoints. Each dot represents the mean expression per individual. Box: median and IQR; whiskers: 1.5×IQR. q-values were computed with two-sided Wilcoxon rank-sum tests comparing each disease stage to HD and corrected using the BH method. (D) Box plot comparing APRIL-responsive gene module scores in post-vaccination B cells between HD (n=18) and SMM (n=18). The module comprises 15 literature-derived APRIL target genes spanning NF-κB pathway components (NFKB1, NFKB2, RELB, NFKBIA), pro-survival factors (MCL1, BCL2), adhesion molecules (CD44, ICAM1), and immunomodulatory mediators (CCL3, CCL4, VEGFA, IL10, CD274, TGFB1, CXCL8). Each dot represents the mean module score per individual. Box: median and IQR; whiskers: 1.5×IQR. The p-value was computed with a two-sided Mann-Whitney U test. (E) Box plot comparing the same APRIL-responsive gene module scores in bone marrow plasma cells from an independent cohort (GSE193531^36^), comparing normal bone marrow (NBM, n=9) and SMM (n=12). Each dot represents the mean module score per sample. Box: median and IQR; whiskers: 1.5×IQR. The p-value was computed with a two-sided Mann-Whitney U test. (F) Schematic illustrating the proposed mechanistic model of APRIL consumption (created with BioRender). (1) Expanding clonal plasma cells increasingly sequester circulating APRIL. (2) Long-lived plasma cells are progressively deprived of this critical survival signal. (3) Long-term humoral immunity is compromised as normal antibody-secreting cells lose APRIL-dependent maintenance.

To dissect the cellular mechanisms underlying immune dysfunction in myeloma precursors, we performed single-cell RNA sequencing (scRNA-seq) on peripheral blood mononuclear cells from 114 individuals across the disease spectrum (HD n=25, MGUS n=19, SMM n=49, MM n=21), profiling a total of 1,109,633 cells after quality control (Figure 3A; **Supplementary Table 2**). Unsupervised clustering and manual annotation resolved 37 distinct cell populations spanning B cells, T cells, NK cells, monocytes, and dendritic cells (Figure 3B; **Supplementary Figure 1**).

We first asked whether reduced APRIL levels in the plasma of patients with SMM could reflect decreased expression of APRIL by myeloid cells, the primary source of APRIL in peripheral blood. Analysis of *TNFSF13* transcript levels in monocytes and dendritic cells revealed no significant reduction between HD, MGUS, and SMM at either pre- or post-vaccination timepoints (two-sided Wilcoxon rank-sum test with Benjamini-Hochberg correction, all q>0.29; Figure 3C). While transcript levels do not directly measure protein secretion, the absence of a transcriptional deficit suggests that reduced circulating APRIL in SMM is more likely attributable to increased consumption rather than impaired expression. To evaluate the downstream effects of APRIL depletion on normal B cells, we curated a 15-gene signature based on established APRIL-responsive targets (**Supplementary Table 3**). Because APRIL signals through BCMA and TACI to trigger both canonical and non-canonical NF-κB pathways^27–34^, our module incorporates core pathway components (*NFKB1, NFKB2, RELB, NFKBIA*) and several targets validated by Tai *et a*l. as upregulated by APRIL-BCMA signaling. These include key pro-survival factors (*MCL1, BCL2*), adhesion molecules (*CD44, ICAM1*), and various immunomodulatory mediators (*CCL3, CCL4, VEGFA, IL10, CD274, TGFB1, CXCL8*)^25^. To independently validate this module, we analyzed RNA-seq data from human plasmablasts stimulated ex vivo with multimeric APRIL (GSE173644)^35^. Twelve of 15 module genes were significantly upregulated at peak transcriptional response (120 minutes post-stimulation; paired t-test, FDR < 0.05), with the remaining genes showing concordant positive trends across the stimulation time course (**Supplementary Figure 2**). Scoring this APRIL-responsive gene module in normal B cells post-vaccination revealed that patients with SMM had significantly reduced expression compared to HD (p=0.006; Figure 3D), with 8 out of the 15 genes in the signature also individually downregulated in SMM (q<0.05; **Supplementary Figure 3**). To validate these findings in an independent cohort, we analyzed normal bone marrow plasma cells from the Boiarsky *et al*. single-cell dataset (GSE193531)^36^, which profiled 9 healthy donors and 12 SMM patients, and differentiated between malignant and normal plasma cells. Scoring cells for the same APRIL-responsive gene module, we observed significantly reduced expression in normal plasma cells from patients with SMM compared to HD (p=0.0095; Figure 3E), confirming that APRIL signaling deficiency is a reproducible feature in normal antibody-producing cells of patients with myeloma precursors.

Together, these data support a mechanistic model in which expanding pre-malignant plasma cells progressively sequester circulating APRIL (Figure 3F). The resulting depletion of this critical cytokine impairs key survival transcriptional programs in normal B cells and plasma cells and may ultimately compromise long-term humoral immunity.

### Impaired *de novo* T cell expansion and activation following antigenic challenge in myeloma precursors

**Figure 4.**
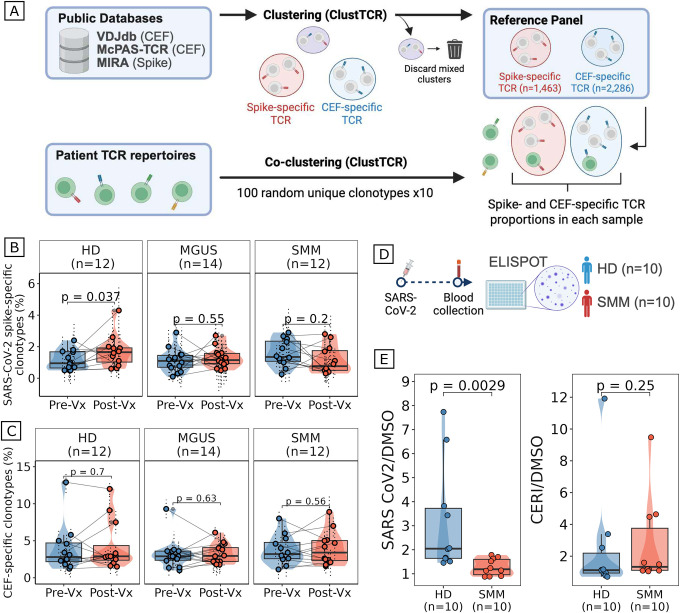
Impaired *de novo* T cell expansion and activation following antigenic challenge in myeloma precursors. (A) Schematic of the computational workflow for in silico identification of antigen-specific T cells (created with BioRender). Spike-specific (n=1,463 clusters) and CEF-specific (n=2,286 clusters; recognizing CMV, EBV, and influenza epitopes) TCR reference panels were built from public databases (VDJdb, McPAS-TCR, MIRA) using ClusTCR. Patient TCR repertoires were co-clustered with references using a bootstrapped sampling strategy (100 random unique clonotypes × 10 iterations per sample) to estimate antigen-specific proportions. Mixed clusters containing both spike- and CEF-specific TCRs were discarded. (B) Box plots, violin plots, and scatter plots of the proportion of TCR clonotypes mapping to spike-specific clusters (y-axis) pre- and post-vaccination in HD (n=12), patients with MGUS (n=14), and patients with SMM (n=12). Each dot represents one individual; paired samples are connected by lines. Violin outline width represents density. Box: median and IQR; whiskers: 1.5×IQR. P-values were computed with two-sided paired Wilcoxon signed-rank tests. (C) Box plots, violin plots, and scatter plots comparing the proportion of CEF-specific (CMV, EBV, influenza) TCR clonotypes (y-axis, %) pre- and post-vaccination in HD (n=12), patients with MGUS (n=14), and patients with SMM (n=12). Each dot represents one individual; paired samples are connected by lines. Violin outline width represents density. Box: median and IQR; whiskers: 1.5×IQR. P-values were computed with two-sided paired Wilcoxon signed-rank tests. No significant changes were observed in any group (HD p=0.70; MGUS p=0.63; SMM p=0.56), confirming that SARS-CoV-2 vaccination does not alter pre-existing memory T cell responses to unrelated viral antigens. (D) Schematic of the IFN-γ ELISPOT experimental design (created with BioRender). Peripheral blood T cells from an independent cohort of HD (n=10) and patients with SMM (n=10) who had received two doses of a SARS-CoV-2 mRNA vaccine were stimulated with SARS-CoV-2 spike protein peptides or CERI peptides (CMV, EBV, RSV, influenza) as a control. (E) box plots, violin plots, and scatter plots of IFN-γ production measured by ELISPOT following stimulation with spike peptides (left) or CERI peptides (right), normalized to DMSO control. Each dot represents the mean of two replicates from one individual. Violin outline width represents density. Box: median and IQR; whiskers: 1.5×IQR. P-values were computed with two-sided Wilcoxon rank-sum tests.

To assess the quality of T cell responses to vaccination, we performed single-cell TCR sequencing on 179 pre- and post-vaccination samples from 82 individuals (HD n=21, MGUS n=19, SMM n=42). These repertoires were then analyzed using a custom computational workflow designed to identify and track the proportion of antigen-specific TCR clonotypes. Specifically, we used ClusTCR to build a reference panel of spike-specific TCRs (n=1,463 clusters) and CEF-specific TCRs (n=2,286 clusters; recognizing CMV, EBV, and influenza epitopes) from public databases, and co-clustered patient TCR repertoires with these references to estimate antigen-specific proportions (Figure 4A; **Supplementary Table 3**). Healthy donors showed a significant increase in spike-specific clonotypes following vaccination (p=0.037), as expected, but no such increase was observed in patients with MGUS (p=0.55) or SMM (p=0.2) (Figure 4B). As expected, CEF-specific clonotypes remained stable across all groups (all p>0.5; Figure 4C), suggesting that our observation pertained specifically to vaccination-induced T cells.

To functionally validate the observed defect, we performed IFN-γ ELISPOT assays on an independent cohort of HD (n=10) and SMM (n=10) patients stimulated with SARS-CoV-2 spike peptides or CERI peptides (CMV, EBV, RSV, influenza) as a control (Figure 4D). Concordant with our *in-silico* analysis of spike-specific T cells, SMM patients showed markedly reduced spike-specific T cell activation following stimulation compared to HD (p=0.0029), while responses to CERI peptides were comparable between groups (p=0.25, Figure 4E). Taken together with our prior results, these findings suggest that patients with myeloma precursors have a general defect in B cell and T cell immunity post-vaccination, which may have important implications for cancer vaccine design and immunotherapy strategies in this population.

### Blunted innate immune activation and cytokine responses to vaccination in myeloma precursors

**Figure 5.**
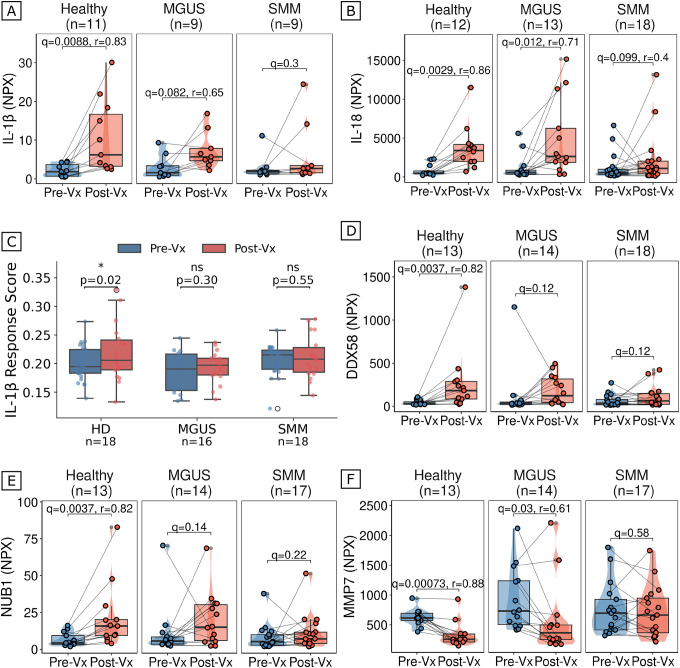
Blunted innate immune activation and cytokine responses to vaccination in myeloma precursors. (A) Paired pre- and post-vaccination peripheral blood plasma IL-1β protein levels (y-axis, Olink NPX) in HD (n=11), patients with MGUS (n=9), and patients with SMM (n=9). Each dot represents one individual; paired samples are connected by lines. Violin outline width represents density. Box: median and IQR; whiskers: 1.5xIQR. q-values were computed with two-sided paired Wilcoxon signed-rank tests and corrected using the BH method across disease groups (3 tests per cytokine). Effect sizes are reported as rank-biserial correlation (r) for comparisons reaching q<0.1. HD showed robust IL-1β induction post-vaccination (q=0.0088, r=0.83), MGUS showed an attenuated response (q=0.082, r=0.65), while SMM failed to mount a significant response (q=0.3). (B) Paired pre- and post-vaccination peripheral blood plasma IL-18 protein levels (y-axis, Olink NPX) in HD (n=12), patients with MGUS (n=13), and patients with SMM (n=18). Plot elements and statistical methods as in (A). HD: q=0.0029, r=0.86; MGUS: q=0.012, r=0.71; SMM: q=0.099, r=0.4. (C) IL-1β response gene signature scores in pre-vaccination (blue) and post-vaccination (red) immune cells from scRNA-seq data in HD (n=18), MGUS (n=16), and SMM (n=18). The IL-1β response signature comprises 1,475 genes derived from a published dictionary of immune responses to cytokines at single-cell resolution39. Each dot represents the mean signature score per individual. Box: median and IQR; whiskers: 1.5xIQR. P-values were computed with two-sided Wilcoxon rank-sum tests comparing pre- and post-vaccination within each group. *p<0.05; ns, not significant. (D) Paired pre- and post-vaccination peripheral blood plasma DDX58 (RIG-I) protein levels (y-axis, Olink NPX) in HD (n=13), patients with MGUS (n=14), and patients with SMM (n=18). Plot elements and statistical methods as in (A). HD showed significant post-vaccination increase (q=0.0037, r=0.82); neither MGUS (q=0.12) nor SMM (q=0.12) reached significance. (E) Paired pre- and post-vaccination peripheral blood plasma NUB1 protein levels (y-axis, Olink NPX) in HD (n=13), patients with MGUS (n=14), and patients with SMM (n=17). Plot elements and statistical methods as in (A). HD: q=0.0037, r=0.82; MGUS: q=0.14; SMM: q=0.22. (F) Paired pre- and post-vaccination peripheral blood plasma MMP7 protein levels (y-axis, Olink NPX) in HD (n=13), patients with MGUS (n=14), and patients with SMM (n=17). Plot elements and statistical methods as in (A). HD: q=0.00073, r=0.88; MGUS: q=0.03, r=0.61; SMM: q=0.58. Across all five innate markers, effect sizes followed a consistent gradient: large in HD (r=0.82–0.88), moderate in MGUS where significant (r=0.61–0.71), and absent in SMM.

Having established that both humoral and cellular arms of the adaptive immune response are impaired in patients with myeloma precursors, we next investigated whether defects in innate immunity might underlie the observed impairment of adaptive immune responses. The inflammasome-processed cytokines, IL-1β and IL18, are critical for optimal vaccine immunogenicity, particularly for mRNA vaccines, where higher post-vaccination concentrations of pro-inflammatory cytokines have been associated with enhanced antibody responses^37^. To characterize post-vaccination inflammatory cytokine dynamics, we analyzed paired pre- and post-vaccination plasma from our Olink proteomics experiment (Figure 5A). HD showed significant increases in both IL-1β (q=0.0088, r=0.83) and IL-18 (q=0.0029, r=0.86) post-vaccination, with large effect sizes consistent with robust inflammasome activation. By contrast, MGUS patients showed attenuated responses (IL-1β: q=0.082, r=0.65; IL-18: q=0.012, r=0.71), while SMM patients failed to mount a significant response for either cytokine (IL-1β: q=0.3; IL-18: q=0.099), indicating a progressive blunting of innate immune activation with advancing disease (Figure 5B). To validate this observation at the transcriptional level, we leveraged a published dictionary of immune responses to cytokines at single-cell resolution^38^ and scored a comprehensive IL-1β response gene signature (1,475 genes) across all immune cell subsets in our scRNA-seq data. Consistent with the proteomic findings, healthy donors showed a significant increase in IL-1β response post-vaccination (p=0.024), whereas neither MGUS (p=0.30) nor SMM (p=0.55) patients showed evidence of IL-1β-driven transcriptional activation (Figure 5C).

**Figure 6.**
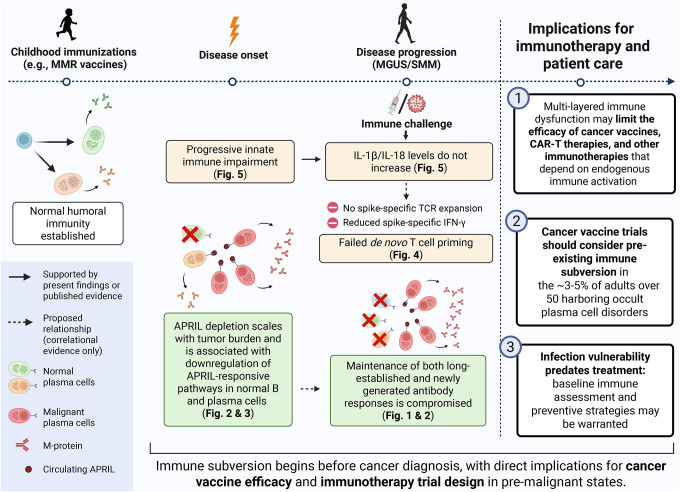
Multilayered immune dysfunction in myeloma precursors compromises durable immunity with implications for cancer immunotherapy. Graphical summary integrating findings from Figures 1–5 (created with BioRender). The timeline progresses from left to right: childhood immunizations establish normal humoral immunity (left), which is progressively eroded following disease onset and during disease progression in MGUS/SMM (center), with implications for cancer immunotherapy and susceptibility to infections (right). Three converging axes of immune dysfunction are depicted. (Bottom) APRIL depletion scales with tumor burden and is associated with downregulation of APRIL-responsive pathways in normal B cells and plasma cells (Figures 2 and 3), compromising maintenance of both long-established and newly generated antibody responses (Figures 1 and 2). (Top) Progressive innate immune impairment results in failure of IL-1b and IL-18 levels to increase following immune challenge (Figure 5). (Middle) Failed de novo T cell priming is evidenced by absent spike-specific TCR clonal expansion and reduced spike-specific IFN-gamma production (Figure 4). Together, this multi-layered immune dysfunction may limit the efficacy of cancer vaccines, CAR-T therapies, and other immunotherapies that depend on endogenous immune activation, while also compromising protective immunity against common pathogens. These findings suggest that immunotherapy trials and infection management strategies should account for pre-existing immune subversion in the estimated 3–5% of adults over 50 who harbor occult plasma cell disorders. Solid arrows indicate relationships supported by present findings or published evidence; dashed arrows indicate proposed relationships supported by correlational evidence only.

In addition to IL-1β and IL18, three other innate immune proteins from the Olink panel showed significant differences in vaccination response between patients and HD. Specifically, DDX58 (RIG-I), a pathogen recognition receptor that can trigger inflammasome assembly and type I interferon signaling^39,40^, and NUB1, an interferon-inducible negative regulator of NEDD8 involved in pro-inflammatory cytokine signaling^41–43^, both showed large post-vaccination increases in HD (q=0.0037, r=0.82 for both) but failed to reach significance in either MGUS or SMM patients (Figure 5D–E). MMP7, a metalloproteinase involved in innate immune defense and tissue remodeling during inflammation^40^, similarly increased in HD (q=7.3×10–4, r=0.88) and MGUS (q=0.03, r=0.61) but remained unchanged in SMM (q=0.58) (Figure 5F). Notably, the effect sizes for all five innate markers followed a consistent gradient, with large effects in HD (r=0.82–0.88), moderate effects in MGUS (r=0.61–0.71 where significant), and absent responses in SMM, mirroring the disease-stage-dependent pattern observed in antibody responses. Taken together, our findings reveal broad immune dysfunction in patients with myeloma precursors spanning both adaptive and innate compartments, with innate immune blunting that may operate upstream of the adaptive defects we observe (Figure 6).

## DISCUSSION

Immune dysfunction in the clinically silent precursor phase of myeloma remains incompletely defined. Previous studies have described individual immune abnormalities in these patients, but it has remained unclear whether these abnormalities arise from defective generation of immunity, defective maintenance of immunity, or both. Here, by combining SARS-CoV-2 vaccination as a standardized immunologic probe with MMR serology as a readout of long-established immunity in a prospectively followed cohort spanning the full MGUS-to-MM spectrum, we show that multilayered immune dysfunction is already established during the precursor phase. This dysfunction spans humoral, cellular and innate compartments, compromises both the generation of new immunity and the maintenance of protection acquired over a lifetime, and scales with disease burden. These findings have direct implications for the design of immunotherapies targeting early-stage disease and for clinical management of infection risk in the growing population of individuals diagnosed with myeloma precursor conditions.

The emergence of cancer vaccines as a therapeutic modality has generated substantial enthusiasm, yet their efficacy depends on an immune system capable of mounting coordinated responses to tumor antigens. Our findings show that immune erosion is already present in the clinically silent precursor phase, likely preceding overt diagnosis by years, raising important questions about the immunologic fitness of patients who are increasingly being considered for early therapeutic intervention, including cancer vaccination^15^. Beyond immunotherapy, the erosion of humoral immunity documented here has immediate clinical relevance: individuals with myeloma precursor conditions may lose protective antibody levels against common pathogens even before receiving cytotoxic therapy. Infections are a leading cause of morbidity and mortality across the myeloma disease spectrum^14,44^, and our data suggest that this vulnerability begins in the precursor phase and is driven by disease biology rather than treatment-related immunosuppression. This distinction is clinically important, as it suggests that infection surveillance and preventive strategies, including assessment of vaccine-derived immunity, may need to be implemented earlier in the disease course than is currently routine.

We found that patients with MGUS and SMM exhibit accelerated loss of humoral immunity affecting both newly induced vaccine responses and decades-old immunity to childhood pathogens such as mumps and rubella. The erosion of long-standing humoral memory points to a fundamental defect in maintenance of long-lived plasma cells, rather than solely impaired de novo antibody generation.

A central regulator of plasma cell survival is the APRIL–BCMA signaling axis. APRIL, produced predominantly by myeloid cells, binds BCMA on plasma cells to provide essential survival signals that sustain long-lived plasma cells within the bone marrow niche and thereby maintain durable humoral immunity after infection or vaccination^27^. Because BCMA is expressed on both normal and malignant plasma cells, these populations may compete for a finite supply of APRIL within the marrow microenvironment. Our data support a model in which expanding clonal plasma cells progressively deplete circulating APRIL. The strong inverse association between tumor burden and plasma APRIL levels, the absence of an apparent transcriptional defect in myeloid APRIL production, and the downregulation of APRIL-responsive survival pathways in patient B cells, validated in an independent cohort, collectively support this interpretation.

Our multi-omic analysis further shows that immune dysfunction in myeloma precursor conditions extends well beyond humoral immunity. Patients failed to expand spike-specific T cell clonotypes despite repeated antigen exposure through vaccination, indicating a defect in de novo cellular immune priming. This observation is particularly relevant to cancer vaccines, which depend on induction of T cell responses against previously unrecognized tumor neoantigens^45^. These data raise the possibility that patients with myeloma precursor conditions may be intrinsically less capable of mounting the de novo T cell responses that such vaccines are designed to elicit. More broadly, our findings support incorporation of baseline immune fitness assessments into vaccine-based immunotherapy trials in hematologic malignancies and suggest that combination approaches designed to enhance T cell priming, including checkpoint blockade or cytokine-based adjuvants, may be needed to overcome this deficit.

The progressive blunting of innate immune activation adds a further layer to this immune dysfunction. Healthy donors mounted robust IL-1β and IL-18 responses after vaccination, consistent with appropriate inflammasome activation, whereas these responses were progressively attenuated across the MGUS-to-SMM spectrum. This pattern parallels observations in severe infection and sepsis^46^, where innate immune dysfunction compromises downstream adaptive responses, and in the tumor microenvironment, where tumor-imposed innate immune suppression is increasingly recognized as a barrier to immunotherapy^47^. The broad attenuation of innate activation suggests that adjuvants capable of providing inflammasome-independent inflammatory signals, such as STING agonists or TLR ligands^48,49^, could help compensate for deficient endogenous IL-1β and IL-18 production and thereby improve immune priming in this setting.

Limitations of this study include the observational design, which precludes definitive causal inference; incomplete mechanistic resolution of how clonal plasma cells deplete systemic APRIL; and the possibility that unmeasured host factors, including age, comorbidities or prior pathogen exposure, may also influence the immune phenotypes observed. In addition, although the SARS-CoV-2 vaccine provided a controlled and clinically relevant antigenic challenge, responses to this platform may not fully capture responses to other vaccines or immunotherapies. Finally, although our findings were supported in an independent cohort for selected mechanistic analyses, further validation in external populations and in other precursor states will be important to define the generalizability of these observations.

Taken together, our findings provide evidence that long-standing humoral memory can be eroded in a precancerous condition and support a mechanistic framework linking precursor disease biology to progressive loss of immune homeostasis. This mechanism may contribute to the increased susceptibility to bacterial and viral infections observed across the myeloma disease spectrum, as the same defect in plasma cell maintenance that erodes vaccine-derived immunity would be expected to compromise pathogen-specific antibody protection more broadly. The clinical implications extend beyond immunotherapy to routine management of patients with precursor conditions: our findings suggest that infection prevention strategies, reassessment of vaccination schedules and monitoring of protective antibody titers may need to be considered even in the absence of symptoms or treatment. As cancer vaccines and other immunotherapies move earlier in the disease trajectory, understanding and addressing this pre-existing immune deficit will be important for realizing their full potential. The IMPACT study, through its prospective design and multi-omic depth, provides a framework for defining immune fitness in early-stage malignancy and for guiding strategies to restore immune competence before therapeutic intervention.

## METHODS

### Study design and patient selection

The IMPACT (IMmune Profiling And COVID-19 Trajectory) study (DFCI Protocol 20–332) is a prospective observational study conducted at Dana-Farber Cancer Institute (DFCI), Boston, MA, designed to characterize immune responses to vaccination in patients with myeloma precursor conditions and matched controls. Participants were recruited from three prospectively followed clinical cohorts at DFCI: (1) the PCROWD study (NCT02269592; DFCI Protocol 14–174), a national tissue-banking study of patients with precursor hematologic malignancies initiated in 2014 with over 2,900 participants enrolled; (2) the PROMISE study (NCT03689595; DFCI Protocol 18–370), a national screening study for monoclonal gammopathies funded by Stand Up To Cancer, conducted in collaboration with Dana-Farber, Johns Hopkins, Mayo Clinic, Harvard T.H. Chan School of Public Health, the Broad Institute, and Quest Diagnostics; and (3) the CureCloud study (NCT03657251; DFCI Protocol 20–044), a collaborative study with the Multiple Myeloma Research Foundation (MMRF) enrolling patients with overt multiple myeloma. Enrollment in one of these three cohorts was a prerequisite for IMPACT participation. Baseline sociodemographic and clinical data collected in the recruitment cohorts were shared with IMPACT to minimize redundant data collection. The study was approved by the Dana-Farber/Harvard Cancer Center Institutional Review Board (Protocol 20–332), and all participants provided written informed consent for the IMPACT study, in addition to consent for their respective recruitment cohort.

The study enrolled 731 individuals across the disease spectrum: healthy donors (HD; n = 129), patients with monoclonal gammopathy of undetermined significance (MGUS; n = 249), patients with smoldering multiple myeloma (SMM; n = 273), and patients with overt multiple myeloma (MM; n = 80) (**Supplementary Table 1**). HD were individuals enrolled in PCROWD or PROMISE who screened negative for monoclonal gammopathy. Participants were enrolled between November 2020 and April 2022. Inclusion criteria were: age >=18 years; for patients, a confirmed diagnosis of MGUS, SMM, or MM according to International Myeloma Working Group (IMWG) criteria; receipt of at least one dose of an FDA-authorized SARS-CoV-2 mRNA vaccine (BNT162b2, Pfizer-BioNTech, or mRNA-1273, Moderna); and availability of at least one pre-vaccination and one post-vaccination blood sample. Exclusion criteria included active systemic anti-myeloma therapy within 3 months of enrollment, immunosuppressive therapy other than low-dose corticosteroids (<=20 mg/day prednisone equivalent), and prior hematopoietic stem cell transplantation.

### Study oversight

The IMPACT study was approved by the Dana-Farber/Harvard Cancer Center Institutional Review Board (Protocol 20–332). All participants provided written informed consent for the IMPACT study, in addition to consent for their respective recruitment cohort (DFCI 14–174, 18–370, or 20–044). The study was conducted in accordance with the Declaration of Helsinki and applicable regulatory requirements. As a prospective observational study, IMPACT is not required to be registered on ClinicalTrials.gov under ICMJE or FDA Section 801 guidelines; the three recruitment cohorts through which participants were identified are registered (NCT02269592, NCT03689595, NCT03657251). This study is reported following the STROBE guidelines for observational studies.

### Sample size

The study enrolled all eligible participants from the recruitment cohorts who met inclusion criteria during the enrollment period, resulting in a total of 731 individuals. Sample sizes for individual assays were determined by specimen availability: scRNA-seq was performed on 114 individuals, scTCR-seq on 82 individuals, plasma proteomics on 51 individuals, and ELISPOT on 20 individuals. No formal power calculation was performed; the study enrolled all eligible participants during the enrollment period. Sample sizes for the recruitment cohorts are described in their respective protocols.

### Sample collection and processing

Peripheral blood samples were collected in EDTA-anticoagulated tubes at protocol-defined timepoints: pre-vaccination (within 2 weeks before the first vaccine dose), and post-vaccination at approximately 2 weeks, 1 month, 2 months, 3 months, and 6 months after the second vaccine dose, with additional samples collected 2–4 weeks after any third dose. Serum was separated by centrifugation and stored at −80 °C until analysis. Peripheral blood mononuclear cells (PBMCs) were isolated by Ficoll density-gradient centrifugation within 4 hours of collection and cryopreserved in 90% fetal bovine serum with 10% DMSO until analysis. All samples were processed and stored at the Ghobrial Laboratory, DFCI.

### ELISA assay to detect IgG antibodies against SARS-CoV-2

Peripheral blood plasma samples were obtained longitudinally from individuals before and after SARS-CoV-2 vaccination, yielding a total of 1,940 samples from 731 participants. The cohort included individuals with monoclonal gammopathy of undetermined significance (MGUS; n = 249), smoldering multiple myeloma (SMM; n = 273), overt multiple myeloma (MM; n = 80), and healthy donors (HD; n = 129). Of the 731 participants, vaccine type information was available for 650 (89%). Among these, 350 received BNT162b2 (Pfizer-BioNTech) and 300 received mRNA-1273 (Moderna). Spike-specific humoral responses were quantified using an enzyme-linked immunosorbent assay (ELISA) to detect anti–SARS-CoV-2 spike protein IgG antibodies, adapted from previously reported methods. Briefly, high-binding ELISA plates (Thermo Fisher, #464718) were coated with recombinant SARS-CoV-2 spike protein in coating buffer (Sigma, #C3041100CAP), followed by sequential washing and blocking. Diluted plasma samples (50 μL per well; dilution buffer containing 1% BSA, 0.05% Tween-20, 140 mM NaCl, and 50 mM Tris, pH 8.0) were incubated for 30 minutes at 37°C. Plates were washed five times and incubated with horseradish peroxidase–conjugated anti-human IgG detection antibody (Bethyl Laboratories, #A80–104P) for 30 minutes at room temperature. After additional washes, signal was developed using TMB substrate (Thermo Fisher, #34029) and stopped with 1 M sulfuric acid. Absorbance was measured at 450 nm with background subtraction at 570 nm using a PHERAstar FSX plate reader. For cross-sectional comparisons (Figures 1B, 1C), post-vaccination samples were stratified by time from the second vaccine dose into early (14–60 days; Figure 1B) and late (61–120 days; Figure 1C) time windows. For participants with multiple samples within a given time window, only the earliest sample was retained to ensure one observation per individual per panel. This filtering yielded subsets of the full cohort for each time window. Post-third-dose titers (Figure 1F) were analyzed separately using samples collected after the third vaccine dose, retaining the earliest sample per participant. ELISA titers were compared across clinical groups using two-sided Wilcoxon rank-sum tests, with multiple hypothesis testing correction performed using the Benjamini-Hochberg method^50^.

### Analysis of spike-specific antibody titer kinetics through longitudinal sampling

Samples serially collected between 2 weeks and 4 months after the second dose of vaccination were included in this analysis (n=75 samples from 35 patients, including 14 patients with MGUS and 21 patients with SMM. Participants received either BNT162b2 (Pfizer-BioNTech; n=18) or mRNA-1273 (Moderna; n=17) vaccines.). To assess whether there were differences in the rate of titer decline between patients with MGUS and SMM, we fitted a linear mixed-effects model with disease-specific intercepts and slopes: *log*(*ELISA*_*Titer*_) = *β*0+ *β*1 * *Disease* + *β*2 * (*Days*_*post-2nd*_ × *Disease*) + *ui*_*individual*_ where β_0_ is the intercept, β_1_ is the fixed effect coefficient for Disease, β_2_ represents the disease-specific slopes (rate of decline over time for each disease group), and u_i_ is the random intercept for individual patients. To test whether the rate of antibody decline differed significantly between disease groups, we compared this model to a constrained model with a common slope across diseases using a likelihood ratio test. For this analysis, only patients with MGUS or SMM who had ≥2 serial samples collected between 14–120 days post-2nd dose were included. This yielded 35 patients (MGUS, n=14; SMM, n=21) with 75 total samples. The serial sampling requirement was necessary for individual slope estimation in the linear mixed-effects model.

### ELISA assay to detect anti-MMR IgG antibodies

Peripheral blood plasma samples were collected from 38 individuals (23 patients with SMM and 15 HD) who were born after 1967 when childhood MMR vaccination became widespread in the United States. ELISA assay to detect anti-Measles, anti-Mumps, and anti-Rubella IgG antibodies were performed using SERION ELISA classic kits (ESR102G, ESR103G, and ESR129G, SERION Diagnostics, Wurzburg, Germany), following the manufacturer’s instruction. Antibody activities in U/ml or IU/ml were determined from the standard curve with the corrected values and compared between groups using two-sided Wilcoxon’s rank-sum tests. P-values were adjusted using the Benjamini-Hochberg approach^50^. Effect sizes for unpaired comparisons were quantified using the rank-biserial correlation (r), computed from the Mann-Whitney U statistic54. Effect sizes are reported for comparisons reaching q<0.1.

### ELISPOT assay to measure IFNγ release upon stimulation with SARS-CoV-2 S antigen peptides

Cryopreserved PBMCs from 10 individuals with SMM and 10 HD were retrieved from liquid nitrogen and rapidly thawed in a 37°C water bath. Cells were washed, pelleted, and resuspended in CTL-Test Medium (Cellular Technology Limited). For each donor, 4 × 10^5^ PBMCs were plated per well in duplicate in 100 μL of medium. Antigen-specific T cell responses were assessed using peptide stimulation followed by IFN-γ ELISPOT. SARS-CoV-2–specific responses were evaluated using a pooled library of 100 spike protein–derived peptides (Mabtech, #3630–1) at a final concentration of 1 μg/mL. Responses to common viral antigens were measured using a composite peptide pool comprising class I–restricted epitopes from cytomegalovirus, Epstein–Barr virus, respiratory syncytial virus, and influenza A virus (CTL-CERI-300, Cellular Technology Limited), also at 1 μg/mL. Negative control wells were treated with vehicle alone (0.3% DMSO). IFN-γ secretion was quantified using a human IFN-γ single-color enzymatic ELISPOT assay (hIFNg-2M/2, Cellular Technology Limited) according to the manufacturer’s instructions. Spot counts were averaged across technical replicates and normalized to paired negative control wells. Antigen-specific responses were expressed as test-to-control ratios and compared between groups using two-sided Wilcoxon rank-sum tests, with multiple testing correction performed using the Benjamini–Hochberg method^50^.

### Inflammatory cytokine measurement in peripheral blood plasma

Plasma cytokine levels were measured using the Olink Proximity Extension Assay technology (Olink Proteomics, Uppsala, Sweden). Data are reported as Normalized Protein expression (NPX) values, which are Olink’s arbitrary unit on a log2 scale, allowing for relative quantification and comparison across samples. A total of 96 samples from 51 participants (19 HD, 14 MGUS, 18 SMM) were used for analysis. Participants received either BNT162b2 (Pfizer-BioNTech; n = 39) or mRNA-1273 (Moderna; n = 12) vaccines. The panel measured 52 cytokines: CCL2, CCL3, CCL4, CCL7, CCL8, CCL11, CCL13, CCL19, CCL20, CLEC4A, CSF1, CSF3, CXCL5, CXCL6, CXCL8, CXCL9, CXCL10, CXCL11, CXCL12, DDX58, EGF, FLT3LG, GZMB, HGF, IFN-γ, IL-1 β, IL-6, IL-7, IL-10, IL-15, IL-17C, IL-18, IL-27, IL4R, IL5RA, ITGB6, LAG3, LTA, MMP1, MMP7, MMP12, NUB1, OLR1, OSM, TGFA, TNF, TNFRSF11A, TNFSF10, TNFSF12, TNFSF13, TNFSF14, and VEGFA. Cross-group comparisons were performed on 52 proteins using two-sided Wilcoxon rank-sum tests across all three groups (HD, MGUS, SMM), generating three pairwise comparisons per protein. P-values were adjusted for multiple comparisons using the Benjamini–Hochberg method across all pairwise comparisons within each timepoint (156 tests per timepoint)^50^. For paired pre- and post-vaccination comparisons within each disease group, two-sided paired Wilcoxon signed-rank tests were used, with p-values adjusted using the Benjamini-Hochberg method across all cytokines tested. Effect sizes for paired comparisons were quantified using the rank-biserial correlation (r), computed from the Wilcoxon signed-rank statistic. Effect sizes are reported for comparisons reaching q<0.1 and interpreted using standard thresholds (small: |r|~0.1; medium: |r|~0.3; large: |r|>=0.5).

### Single-cell sequencing of PBMCs

We performed single-cell RNA sequencing on 224 PBMC samples from 114 participants, including healthy donors (HD, n=25) and patients previously diagnosed with MGUS (n=19), SMM (n=49), or MM (n=21). The SMM cohort included both untreated and previously treated patients at the time of vaccination. Among treated SMM patients (n=27), 23 had completed prior therapy and 4 were on active treatment at the time of vaccination. Prior regimens included daratumumab monotherapy (D-PRISM trial 17–212, n=11) and ixazomib, lenalidomide, and dexamethasone (IRd; trial 16–313, n=11), with remaining patients receiving other regimens (n=5; **Supplementary Table 2**). Samples were collected before vaccination (n=115) and after two doses of vaccination (n=109). Participants received either BNT162b2 (Pfizer-BioNTech; n=66), mRNA-1273 (Moderna; n=42), or Ad26.COV2.S (Janssen; n=8) vaccines. Ninety-one participants provided paired samples before and after two doses of vaccination (HD: 18, MGUS: 17, SMM: 40, MM: 11).

Samples were either collected on site at DFCI or at Quest Diagnostics sites across the country and were subsequently shipped to DFCI overnight for processing. PBMCs were isolated using Ficoll separation or autoMACS (Miltenyi, catalog number 130–115-169) and cryopreserved in FBS supplemented with 10% DMSO. Cryopreserved cells were rapidly thawed in a 37°C water bath, centrifuged at 330 × g for 5 minutes, and washed twice with ice-cold phosphate-buffered saline supplemented with 0.04% ultrapure bovine serum albumin. Following viability recovery and cleanup, cells were loaded onto a Chromium Controller (10x Genomics) for droplet-based single-cell encapsulation, targeting the recovery of approximately 10,000 peripheral blood mononuclear cells per sample, as previously described^13,51^. Single-cell libraries were generated using the Chromium Next GEM Single Cell 5′ Reagent Kit v2 (Dual Index), following the manufacturer’s protocols. Sequencing was performed on a NovaSeq 6000 S4 flow cell at the Broad Institute of MIT and Harvard Genomics Platform (Cambridge, MA).

### Single-cell RNA-sequencing data processing and analysis

Raw sequencing data were processed using Cell Ranger (v6.0.1) to demultiplex FASTQ files and generate gene–cell count matrices. Ambient RNA contamination was removed using CellBender (v0.3.0) with a false positive rate of 0.01 and GPU acceleration. Downstream analyses were performed in Python using Scanpy (v1.10.4). Quality control filtering removed cells with fewer than 200 or more than 5,000 detected genes, fewer than 400 or more than 50,000 total counts, or greater than 15% mitochondrial reads. Genes detected in fewer than 3 cells were excluded. After initial filtering, 1,441,296 cells remained for downstream analysis. Libraries were normalized to 10,000 counts per cell followed by log1p transformation. Highly variable genes were identified using mean-variance modeling (min_mean=0.01, max_mean=3, min_disp=0.3), excluding immunoglobulin genes, ribosomal protein genes, and T cell receptor genes to prevent clonotype-driven clustering. Expression values were scaled with clipping at ±10 standard deviations. Principal component analysis was performed using the arpack solver. Batch effects were corrected using Harmony integration on patient identity with a maximum of 50 iterations. A k-nearest neighbor graph was constructed using 30 neighbors in the Harmony-corrected PCA space, and UMAP embeddings were computed for visualization. Clustering was performed using the Leiden algorithm at resolution 0.5 for initial cell type identification. Putative doublets were identified using Scrublet with an expected doublet rate of 6%. Cells were annotated in two stages: first into major lineages (T cells, B cells, monocytes, dendritic cells, NK cells) based on canonical marker gene expression, then subclustered and annotated into 37 fine-grained populations. After removal of doublets and low-quality clusters, 1,109,633 cells were retained for downstream analysis.

### APRIL-responsive gene module scoring

To assess the downstream transcriptional consequences of APRIL depletion in myeloma precursors, we compiled a module of 15 APRIL-responsive genes from the literature. Genes were selected based on established APRIL-BCMA signaling targets validated by Tai et al.^25^ and known NF-κB transcriptional targets induced upon pathway activation^33^. The module comprised four functional categories: pro-survival factors (MCL1, BCL2), adhesion molecules (CD44, ICAM1), immunomodulatory mediators (CCL3, CCL4, VEGFA, IL10, CD274, TGFB1, CXCL8), and NF-κB pathway components (NFKB1, NFKB2, RELB, NFKBIA). B cells were identified from the scRNA-seq dataset based on Annotation_Level_1 classification and subset to post-vaccination (after 2nd dose) samples from healthy donors (HD) and untreated Patients with SMM (treatment-naive). Module scores were computed using the sc.tl.score_genes() function in Scanpy, which calculates per-cell scores as the mean expression of the gene set minus the mean expression of a reference set of control genes matched for expression level. For patient-level comparisons, module scores were aggregated by computing the mean score across all B cells per patient. Differences between HD and untreated SMM were assessed using two-sided Mann-Whitney U tests on patient-level scores. For individual gene-level analysis, log1p-normalized expression of each of the 15 APRIL-responsive genes was compared between HD and untreated SMM B cells using two-sided Mann-Whitney U tests. P-values were corrected for multiple testing using the Benjamini-Hochberg (BH) method, and adjusted p-values (q-values) below 0.05 were considered significant.

### External validation of the APRIL-responsive gene module (GSE173644)

To validate the 15-gene APRIL-responsive module, we used publicly available RNA-seq data from Stephenson et al. (GEO accession GSE173644)^35^. In this dataset, human plasmablasts generated in vitro (Day 7 post-vaccination) were stimulated with multimeric APRIL (100 ng/ml) and harvested at 0, 30, 60, 120, and 360 minutes (n=4 donors per timepoint). Pre-processed variance-stabilized transformation (VST) normalized expression values were downloaded from GEO. For each gene at each timepoint, we computed the paired difference in expression relative to baseline (0 min) within each donor and performed a paired t-test (two-sided). P-values were corrected for multiple testing using the Benjamini-Hochberg method across the 15 module genes at each timepoint independently. Genes with FDR < 0.05 and positive fold change were considered significantly upregulated. For visualization, expression values were Z-score normalized across timepoints for each gene.

### External validation of APRIL-responsive gene expression in normal bone marrow plasma cells (GSE193531)

To validate the APRIL-responsive gene module findings in an independent cohort, we analyzed a publicly available scRNA-seq dataset of bone marrow plasma cells (GSE193531)^36^. This dataset comprises 29,387 plasma cells from 21 individuals, including 9 healthy donors (normal bone marrow, NBM) and 12 patients with SMM. Raw UMI count matrices and cell-level metadata were downloaded from the Gene Expression Omnibus (GEO). Counts were normalized to 10,000 reads per cell and log1p-transformed using Scanpy. The same 15-gene APRIL-responsive module was scored using sc.tl.score_genes(), and scores were aggregated to the sample level by computing the mean across all cells per sample. Differences between NBM and SMM were assessed using a two-sided Mann-Whitney U test on sample-level scores. Individual gene expression was compared between NBM and SMM plasma cells using two-sided Mann-Whitney U tests with BH correction. To assess cross-dataset concordance, the direction of differential expression (higher in healthy vs. higher in SMM) was compared gene-by-gene between the IMPACT (peripheral blood B cells) and GSE193531 (bone marrow plasma cells) datasets.

### Single-cell TCR-sequencing data processing and analysis

CellRanger vdj (v6.0.1) was used to process V(D)J libraries^52^. Filtered V(D)J contigs were then processed to select a single solution per chain and cell barcode, based on the number of UMIs, as previously described^13,51^. Cells annotated as T lymphocytes and possessing matched V(D)J sequencing data were included in TCR repertoire analyses. For each individual, clonotype information was aggregated across all available samples, aliquots, and longitudinal time points. Clonotypes were redefined in a harmonized manner based on the recurrence of identical complementarity-determining region 3 (CDR3) amino acid sequences across samples, enabling unified tracking of clonal populations.

### Antigen-specific T cell identification and analysis

CDR3β sequences specific to Cytomegalovirus, Epstein-Barr virus, and Influenza (CEF) were collected from VDJdb and McPAS-TCR, while SARS-CoV-2 S spike-specific TCRs were obtained from Adaptive Biotechnologies’ MIRA database^53–55^. These antigen-specific CDR3 sequences were pooled and clustered using ClusTCR’s two-step method^56^. Mixed clusters containing both spike-specific and CEF-specific CDR3βs were discarded as non-specific. Clusters with only spike-specific or CEF-specific CDR3βs were retained and were used as a “reference panel” against which a sample’s TCR repertoire could be tested (**Supplementary Table 4**). To estimate the proportion of spike-specific and CEF-specific TCR clones per sample, we randomly sampled 100 unique clonotypes 10 times and each time, we co-clustered them with the reference panel using ClusTCR’s two-step method^56^. For each random subset, we calculated the proportion of clonotypes that clustered with spike-specific or CEF-specific reference clusters, and subsequently averaged proportions across iterations for each sample. The proportion of spike-specific and CEF-specific clonotypes pre- and post-vaccination were compared using two-sided paired Wilcoxon’s rank-sum tests within each group of individuals. To ensure our analyses were not affected by the shipment-induced immune cell activation, we repeated analyses separating shipped and non-shipped groups of samples and demonstrated no difference in the observed results^56^ (**Supplementary Figure 4**).

### Evaluation of immune response signature to IL1β

To assess IL-1β-induced immune activation following vaccination, we leveraged a curated gene expression signature from the Dictionary of Immune Responses to Cytokines, which identified genes upregulated in response to IL-1β stimulation across immune cell types^38^. The signature comprised 1,475 IL-1β response genes, which were converted from mouse to human gene symbols; of these, 179 genes were detected in our single-cell dataset and used for scoring. For each immune cell population (B cells, T cells, monocytes, dendritic cells, and NK cells), we scored the IL-1β response signature using scanpy’s score_genes function, which calculates the average expression of signature genes relative to a reference set of randomly selected genes with similar expression levels. Signature scores were computed for each cell, then aggregated by calculating the mean score per patient and timepoint within each cell type. Patient-level IL-1β response scores were obtained by averaging across all immune cell types. To compare pre- and post-vaccination IL-1β response signatures, we restricted the analysis to patients with paired samples at both timepoints (HD, n=18; MGUS, n=17; SMM, n=18). For SMM, only treatment-naïve patients were included to match the Olink proteomics cohort. Statistical significance was assessed using two-sided paired Wilcoxon signed-rank tests.

### Statistical analysis

Unless otherwise specified, continuous variables were compared between two groups using two-sided Wilcoxon rank-sum tests (Mann-Whitney U tests) and among three or more groups using Kruskal-Wallis tests followed by pairwise Wilcoxon tests. Paired samples were compared using two-sided Wilcoxon signed-rank tests. Correlations were assessed using Spearman’s rank correlation coefficient for non-parametric data and Pearson correlation coefficient for parametric data. All p-values were adjusted for multiple hypothesis testing using the Benjamini-Hochberg method to control the false discovery rate^50^. Statistical analyses were performed in R (v4.1.3) and Python (v3.9).

## Supplementary Files

This is a list of supplementary files associated with this preprint. Click to download.

- SupplementaryTable1ELISAcohort.csv
- SupplementaryTable3APRILresponsivegenes.csv
- SupplementaryTable4AntigenspecificTCR.csv
- SupplementaryTable2scRNAseqsamplelist.csv
- SupFig4SpikeClonotypesShippedVsNonShipped.png
- SupFig1scRNAseqAnnotation.png
- SupFig3APRILgenesviolins.png
- SupFig2APRILsignaturevalidation.png

## Data Availability

Raw single-cell RNA and TCR sequencing data generated for this study have been deposited in dbGaP (study site pending). Gene expression and TCR clonotype matrices as well as ELISA titers, and Olink proteomics data have been deposited in Zenodo at https://tinyurl.com/IMPACTDFCI (10.5281/zenodo.18989223).

## CODE AVAILABILITY

Code used for downstream analyses is available on GitHub at: https://github.com/ghobriallab/IMPACT_2026.git
